# A Validated CFD Model for Gas Exchange in Hollow Fiber Membrane Oxygenators: Incorporating the Bohr and Haldane Effects

**DOI:** 10.3390/membranes15090268

**Published:** 2025-09-04

**Authors:** Seyyed Hossein Monsefi Estakhrposhti, Jingjing Xu, Margit Gföhler, Michael Harasek

**Affiliations:** 1 Institute of Engineering Design and Product Development, Technische Universität Wien, 1060 Vienna, Austria; jing.xu@tuwien.ac.at (J.X.); margit.gfoehler@tuwien.ac.at (M.G.); 2 Institute of Chemical, Environmental and Bioscience Engineering, Technische Universität Wien, 1060 Vienna, Austria; michael.harasek@tuwien.ac.at

**Keywords:** computational fluid dynamics (CFD), Bohr and Haldane effects, gas exchange, physiological coupling, oxygenator optimization

## Abstract

Chronic respiratory diseases claim nearly four million lives annually, making them the third leading cause of death worldwide. Extracorporeal membrane oxygenation (ECMO) is often the last line of support for patients with severe lung failure. Still, its performance is limited by an incomplete understanding of gas exchange in hollow fiber membrane (HFM) oxygenators. Computational fluid dynamics (CFD) has become a robust oxygenator design and optimization tool. However, most models oversimplify O_2_ and CO_2_ transport by ignoring their physiological coupling, instead relying on fixed saturation curves or constant-content assumptions. For the first time, this study introduces a novel physiologically informed CFD model that integrates the Bohr and Haldane effects to capture the coupled transport of oxygen and carbon dioxide as functions of local pH, temperature, and gas partial pressures. The model is validated against in vitro experimental data from the literature and assessed against established CFD models. The proposed CFD model achieved excellent agreement with experiments across blood flow rates (100–500 mL/min ), with relative errors below 5% for oxygen and 10–15% for carbon dioxide transfer. These results surpassed the accuracy of all existing CFD approaches, demonstrating that a carefully formulated single-phase model combined with physiologically informed diffusivities can outperform more complex multiphase simulations. This work provides a computationally efficient and physiologically realistic framework for oxygenator optimization, potentially accelerating device development, reducing reliance on costly in vitro testing, and enabling patient-specific simulations.

## 1. Introduction

Chronic respiratory diseases (CRDs), with nearly 4 million mortalities annually, rank as the third leading cause of death worldwide [1]. Extracorporeal membrane oxygenation (ECMO) is a life-support technique that supports patients with severe lung dysfunction by maintaining physiological O_2_ and CO_2_ levels in the blood [2]. The oxygenator is the main component of the ECMO circuit, which is composed of thousands of microporous hollow fiber membranes (HFMs), woven into sheets and folded into bundle structures, enabling diffusion of oxygen into the blood and the removal of carbon dioxide. ECMO usage increased significantly during the COVID-19 pandemic, particularly for patients with acute respiratory distress syndrome (ARDS), highlighting its essential role in managing critical respiratory conditions [3]. Despite its clinical benefits, ECMO is associated with significant complications such as hemolysis (rupture of red blood cells due to mechanical shear stress induced by the blood pump and cannulae) [4], thrombosis (clot formation within the circuit) [5], and bleeding due to systemic anticoagulation [6]. These complications contribute to high morbidity and mortality rates, with reported survival ranging from approximately 40–60% for veno-arterial (VA) ECMO and 21–37% for veno-venous (VV) ECMO [7]. Improving clinical results requires a better knowledge of gas exchange dynamics inside the HFM oxygenator. Traditionally, design optimization has been based on labor-intensive and costly experimental prototyping.

In contrast, computational fluid dynamics (CFD) offers a more efficient and cost-effective alternative by simulating blood flow and gas transport within the oxygenator [8]. Moreover, CFD simulations provide deep insight into oxygen and carbon dioxide exchange mechanisms, guiding device development while minimizing the need for significant physical testing. Since research into ECMO technology is expanding, advanced modeling approaches such as using CFD in analyzing and optimizing oxygenator performance are becoming increasingly important.

Many studies have utilized CFD to investigate the effect of various parameters on oxygenator performance, such as fiber arrangement [9,10,11], bundle porosity [12,13], fiber shape [14], hematocrit [13], sweep gas flow rate [15], and thrombosis formation [16,17]. Additionally, several studies have used CFD to validate the oxygen and carbon dioxide transfer rate observed in their experiments [10,12,13,15,17,18,19,20,21]. Some of these studies conducted CFD simulations at the fiber scale, modeling individual fibers in different arrangements with or without the membrane structure, capturing the flow and gas transport around the fibers [9,10,11,12,13,14,18,20,21]. Others performed CFD simulations at the device scale, modeling the oxygenator as a porous medium, capturing the macroscale flow and transport behavior [15,16,17,19].

Zhang et al. introduced a new oxygen transport model considering dissolved oxygen in plasma and the hemoglobin-bound oxygen inside red blood cells. In their approach, the diffusion of the hemoglobin-bound oxygen was neglected due to its low diffusivity, and the membrane fiber bundle was modeled as a porous medium using the Ergun equation [19]. Later, Taskin et al. utilized the same transport equation, modeling the oxygenator at the fiber scale, incorporating the membrane. Oxygen diffusivity within the membrane was calculated using the Knudsen equation [12]. In these studies, oxygen saturation, which plays an essential role in determining the effective diffusivity of oxygen and the overall oxygen transfer rate, was modeled by the Hill equation, assuming a constant value for the half-saturation oxygen partial pressure and the Hill coefficient. The modeling of blood as a two-phase flow, considering plasma and red blood cell phases for simulating oxygen transport, was first introduced by Wright [22]. Later, Keasler et al. adopted a similar approach and validated their CFD model using in vitro experiments on three micro-oxygenators with a staggered fiber configuration under multiple blood flow conditions [18]. The Schiller–Naumann interphase drag model used in their study falsely predicted high red blood cell concentrations at the fiber walls [10], opposite to the formation of a cell-free layer observed experimentally by Bardón et al. [23]. To address this challenge, Focke et al. employed a homogeneous Eulerian–Eulerian approach without modeling mechanical interaction between the phases [10]. In contrast, Poletti et al. used an inhomogeneous Eulerian–Eulerian approach incorporating the Schiller–Naumann drag model with an interphase momentum exchange coefficient [13].

Regarding the carbon dioxide transport, most CFD studies adopt the mathematical model introduced by Svitek and Federspiel [24]. This model accounts for the diffusion of the dissolved CO_2_ and bicarbonate and convection of dissolved CO_2_ and bound CO_2_ (including bicarbonate and CO_2_ bound to hemoglobin). However, the CO_2_ content curve slope was assumed constant in their model. In reality, CO_2_ partial pressure near the fiber wall can be very low, making this assumption invalid in that region. Although the studies above validated their CFD model with in vitro experiments, they relied on assumptions limiting their generalizability. For instance, most of these studies focused exclusively on either oxygen or carbon dioxide transport, without accounting for their physiological interdependence. Therefore, a comprehensively validated CFD model that accurately captures oxygen and carbon dioxide transport remains necessary.

The oxygen saturation curve, which was treated as constant in previous studies, can shift in response to changes in pH, carbon dioxide partial pressure, blood temperature, and 2,3 DPG levels. An increase in pH or a decrease in carbon dioxide partial pressure, temperature, and 2,3 DPG concentration shifts the oxygen saturation curve to the left, increasing hemoglobin’s affinity for oxygen [25]. This physiological phenomenon, the Bohr effect, describes how hemoglobin’s oxygen affinity reduces when carbon dioxide levels increase or pH decreases (i.e., acidity). In the lungs, where carbon dioxide concentration is low and pH is relatively high, hemoglobin exhibits greater oxygen affinity, resulting in higher oxygen saturation. Conversely, in peripheral tissues with higher carbon dioxide levels and lower pH, hemoglobin’s oxygen affinity decreases, facilitating the release of oxygen and thereby reducing oxygen saturation. Similarly, in oxygenators, near the fiber wall where carbon dioxide partial pressure is lower, oxygen saturation increases, reducing the capacity for additional oxygen uptake and thereby decreasing the local oxygen transfer rate [26].

Similarly, oxygen saturation level, pH, and blood temperature influence the carbon dioxide content. This physiological phenomenon is known as the Haldane effect [27]. An increase in oxygen saturation, pH, and temperature results in a rightward shift. As oxygen binds more to hemoglobin, its ability to form carbamino compounds and buffer hydrogen ions is diminished. In the lungs, where oxygen concentration is high, hemoglobin binds with oxygen and releases carbon dioxide and hydrogen ions, helping to remove carbon dioxide during exhalation. Conversely, in peripheral tissues with lower oxygen levels, hemoglobin binds more carbon dioxide and hydrogen ions, promoting carbon dioxide uptake and transport back to the lungs. In oxygenators, near the fiber wall where oxygen saturation is higher, hemoglobin’s ability to carry carbon dioxide decreases, reducing the local carbon dioxide transfer rate. While a few studies have considered these effects in their mathematical models [28,29], most existing CFD studies neglect this physiological interdependence.

This study aims to develop a new CFD model that incorporates both the Bohr and Haldane effects and validate it with existing in vitro experimental data. Specifically, the model is validated using the experimental results from Focke et al., and the predicted results are compared with those from the experiment itself, Focke’s CFD model, Taskin’s model for oxygen transfer [12], and the Svitek and Federspiel model for carbon dioxide transfer [24].

## 2. Materials and Methods

### 2.1. Numerical Domain

**Figure 1:**
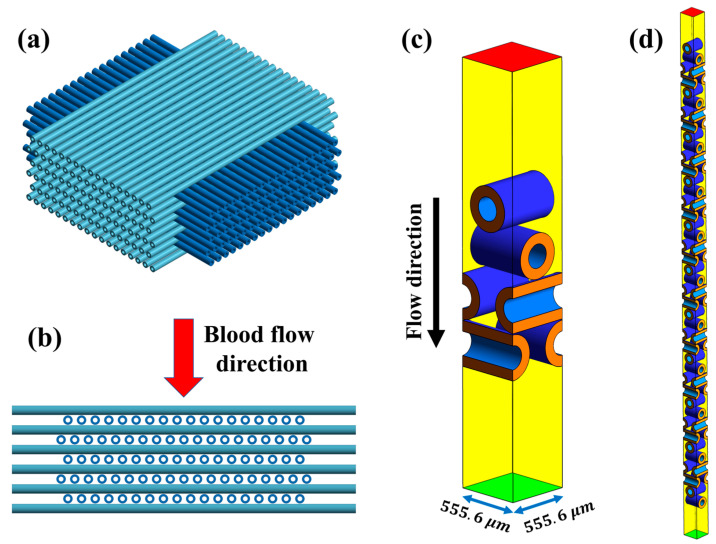
Structure of the 90-degree stacked fiber bundle and corresponding CFD domains. (**a**) Isometric view of the 90-degree stacked fiber bundle in staggered arrangement; (**b**) Side view illustrating the blood flow direction through the bundle; (**c**) CFD domain with four layers of fiber mats used for mesh sensitivity study; (**d**) Full CFD domain representing the entire oxygenator length with 42 layers of staggered fiber mats. Color scheme: red = inlet, green = outlet, yellow and orange = symmetry boundaries for blood and membrane, blue = wall.

In this study, the proposed CFD model is validated against the experimental results from Focke et al. [10], which were obtained using a small oxygenator featuring a 90-degree stacked fiber bundle. Figure 1a,b illustrate the structure of this fiber bundle. Similarly to Focke’s study, in this configuration, every other fiber mat was shifted half a fiber spacing in a staggered arrangement. However, unlike their study, fiber contacts were neglected, and a minimal gap of 10 μm was introduced between adjacent mats. The distance between fibers within each mat was 555.6 μm, corresponding to 18 fibers per centimeter (Oxyplus, Membrana GmbH, Wuppertal, Germany). The hollow fiber membranes were modeled with an outer diameter of 380 μm and an inner diameter of 200 μm. The total length of the experimental oxygenator was 16 mm, corresponding to 42 layers of fiber mats.

As shown in Figure 1c, CFD simulations were initially conducted using a geometry consisting of four layers of fiber mats arranged in a staggered configuration to evaluate mesh sensitivity and determine the optimal mesh. Inlet and outlet extensions of 800 μm were added to the domain to ensure fully developed flow conditions at the boundaries. Despite the approach taken by Focke et al. [10], in which a periodic subdomain was modeled and solved repeatedly to simulate the full oxygenator, the present study modeled the entire geometry directly, consisting of 42 layers of fiber mats (Figure 1d).

### 2.2. Governing Equations, Boundary Conditions, and Simulation Settings

The steady-state flow of blood is modeled by solving the incompressible Navier–Stokes equations,

(1) $$\rho \left(\frac{\partial \vec{v}}{\partial t}+\left(\vec{v}\cdot \nabla \right)\vec{v}\right)=-\nabla p+\mu \nabla^{2}\vec{v}$$

(2) $$\nabla \cdot \vec{v}=0,$$

where ρ is the fluid density in $kg/m^{3}$, $\vec{v}$ is the fluid velocity vector in m/s, p is the pressure in Pa, and μ is the fluid viscosity in Pa.s. These equations represent the conservation of momentum and mass within a differential control volume. Solving the Navier–Stokes equations with appropriate initial and boundary conditions can determine the velocity and pressure fields throughout the discretized computational domain. Ansys Fluent (Ansys, Inc., Canonsburg, PA, USA) performed the CFD simulations that numerically solve the Navier–Stokes equations.

Similar to our previous study [9], blood was modeled as a non-Newtonian fluid using the Carreau-Yasuda viscosity model, with a constant density of 1050 $kg/m^{3}$. Experiments were conducted at blood flow rates of 100, 200, 300, 400, and 500 mL/min, corresponding to superficial velocities of 0.34, 0.68, 1.02, 1.36, and 1.70 cm/s, and Reynolds numbers of 0.7, 1.4, 2.1, 2.8, and 3.5, respectively, indicating laminar flow conditions.

A constant velocity corresponding to the specified superficial velocities was applied at the inlet, and a constant gauge pressure of 0 Pa was imposed at the outlet. Symmetric boundary conditions were applied to the sides of the domain, representing a repetitive fiber arrangement and flow pattern. Moreover, the inlet and inner membrane walls were assigned constant partial pressures. As this study employs the Kelman subroutine [30] to compute oxygen saturation, unlike the Siggaard-Andersen model [31] used by Focke et al. [10], slight adjustments were made to match the inlet saturation and maintain the same partial pressure difference. Specifically, an inlet oxygen saturation of 0.65 was achieved using a partial pressure of 35.9 mmHg, instead of the 41.2 mmHg used by Focke et al [10]. An oxygen partial pressure of 707.7 mmHg was applied at the inner membrane wall to preserve the original partial pressure difference across the membrane, replacing the 713 mmHg used in the earlier study. Carbon dioxide partial pressures remained unchanged, with 44 mmHg imposed at the inlet and zero mmHg at the inner membrane wall.

Similar to the approach used by Taskin et al. [12], the membrane region is explicitly modeled in this study. Gas transport through the porous fiber wall is assumed to follow the Knudsen diffusion mechanism, which dominates in small pores where gas molecules primarily collide with the pore walls. The effective diffusion coefficients for oxygen and carbon dioxide within the membrane were calculated based on membrane permeation properties, including tortuosity, effective porosity, and effective pore length, using the following relationship:

(3) $$D_{i}=K_{m}\frac{\tau }{\varepsilon }\;h\;\left(\frac{T}{T_{0}}\right)\;P_{0}.$$

where $K_{m}$ is the membrane permeation adopted from the study by Eash et al. [32] for Membrana fibers. The permeation values are $1.08\;\text{×}\;10^{-3}\;cm^{3}/s/cm^{2}/\mathrm{cmHg}$ for oxygen and $1.40\;\text{×}\;10^{-3}\;cm^{3}/s/cm^{2}/\mathrm{cmHg}$ for carbon dioxide. The tortuosity (τ) is estimated to be 4. The effective pore diameter and porosity of the PMP hollow fibers are reported in the literature to be 249 nm [33] and 0.5 [34], respectively. T is the temperature, assumed to be 37 °C or 310 K. $T_{0}$ and $P_{0}$ represent standard temperature and pressure, defined as 298 K and 760 mmHg, respectively.

The SIMPLE algorithm was used for pressure-velocity coupling, discretizing pressure using the PRESTO scheme and momentum using the QUICK scheme. A convergence criterion of $10^{-5}$ was set for all variables.

### 2.3. Oxygen Transport Simulations

The steady-state transport of oxygen is modeled by solving the advection-diffusion-reaction equation:

(4) $$\vec{v}\;\cdot \;\nabla P_{O_{2}}=D_{eff,\;O_{2}}\;\nabla^{2}P_{O_{2}}+\;S_{P_{O_{2}}}$$

where $P_{O_{2}}$ is the oxygen partial pressure (mmHg), $D_{eff,O_{2}}$ is the effective diffusivity of oxygen in blood ($m^{2}/s$), and $S_{P_{O_{2}}}$ is the source term for oxygen partial pressure (mmHg/s). The effective diffusivity and source term are defined in Equations (5) and (6), respectively. The derivation of these expressions is provided in Appendix A.

(5) $$D_{eff,O_{2}}=\frac{D_{O_{2}}\;\alpha_{O_{2}}+D_{HbO_{2}}k_{O_{2}}\;Hb\frac{\partial S_{O_{2}}}{\partial P_{O_{2}}}}{\alpha_{O_{2}}+k_{O_{2}}\;Hb\frac{\partial S_{O_{2}}}{\partial P_{O_{2}}}}$$

(6) $$S_{P_{O_{2}}}=\frac{D_{HbO_{2}}k_{O_{2}}\;Hb\frac{\partial^{2}S_{O_{2}}}{\partial P_{O_{2}}^{2}}\;}{\alpha_{O_{2}}+k_{O_{2}}\;Hb\frac{\partial S_{O_{2}}}{\partial P_{O_{2}}}}\;|\nabla P_{O_{2}}{|}^{2}$$

where $D_{O_{2}}$ is the diffusivity of oxygen in blood ($m^{2}/s$), $\alpha_{O_{2}}$ is the solubility of oxygen in blood ($mL_{O_{2}}/mL_{\mathrm{blood}}/\mathrm{mmHg}$), $D_{HbO_{2}}$ is the diffusivity of oxyhemoglobin ($m^{2}/s$), $k_{O_{2}}$ is the oxygen-carrying capacity of hemoglobin, also known as the Hüfner coefficient ($mL_{O_{2}}/g_{\mathrm{Hb}}$), Hb is the total hemoglobin concentration ($g_{\mathrm{Hb}}/mL_{\mathrm{blood}}$), and $S_{O_{2}}$ is the oxygen saturation of hemoglobin (dimensionless). Since the second derivative of $S_{O_{2}}$ with respect to $P_{O_{2}}$ is very small, the source term ($S_{P_{O_{2}}}$) is assumed to be 0. The physical constants used for oxygen and carbon dioxide transport calculations are summarized in Table 1.

As mentioned earlier, oxygen saturation ($S_{O_{2}}$) in this study is calculated using the Kelman subroutine [30]. Unlike the Hill equation [35], which defines saturation based on the oxygen partial pressure at 50% hemoglobin saturation ($P_{50}$) and an exponent n, Kelman’s formulation expresses oxygen saturation only as a function of adjusted oxygen partial pressure. To account for the Bohr effect, an adjusted oxygen partial pressure was defined as a function of the actual oxygen partial pressure, blood temperature, pH, and carbon dioxide partial pressure. Equations (7) and (8) present the Kelman subroutine used for calculating oxygen saturation.

(7) $$P_{O_{2},adj}=P_{O_{2}}\times 10^{0.024\;\left(37-T\right)+0.40\;\left(pH-7.4\right)+0.06\;log(40/P_{CO_{2}})\;}$$

(8) $$S_{O_{2}}=\frac{a_{1}{\;P}_{O_{2},adj}+a_{2}{\;P}_{O_{2},adj}^{2}+a_{3}{\;P}_{O_{2},adj}^{3}+P_{O_{2},adj}^{4}}{a_{4}+a_{5}\;P_{O_{2},adj}+a_{6}\;P_{O_{2},adj}^{2}+a_{7}\;P_{O_{2},adj}^{3}+P_{O_{2},adj}^{4}}$$

where $a_{1}$ to $a_{7}$ are empirically derived coefficients with the following values: $a_{1}=-8532.229$, $a_{2}=2121.401$, $a_{3}=-67.07399$, $a_{4}=935960.9$, $a_{5}=-31346.26$, $a_{6}=2396.167$, and $a_{7}=-67.10441$. To calculate oxygen saturation, the blood pH must first be determined. Oxygen saturation is then computed based on the blood temperature, pH, and partial pressure of carbon dioxide. The method used for pH calculation is described in the following section.

The total concentration of oxygen in blood, comprising both dissolved oxygen in plasma and oxygen bound to hemoglobin, is calculated as follows:

(9) $$C_{O_{2}}=\;\alpha_{O_{2}}\;P_{O_{2}}+\;k_{O_{2}}\;Hb\;S_{O_{2}}$$

Therefore, the oxygen transfer rate (${\dot{m}}_{O_{2}}$) is calculated using Equation (10), based on the mass-weighted averages of oxygen partial pressure and oxygen saturation at the inlet and outlet.

(10) $${\dot{m}}_{O_{2}}=Q_{blood}\times 10^{3}\times \left(\alpha_{O_{2}}\;\left(P_{O_{2},out}-P_{O_{2},in}\right)+k_{O_{2}}\;Hb\;\left(S_{O_{2},out}-S_{O_{2},in}\right)\right)$$

where $Q_{blood}$ is the blood flow rate ($L_{\mathrm{blood}}/\min$).

### 2.4. Carbon Dioxide Transport Simulations

Similar to oxygen transport, the steady-state transport of carbon dioxide is modeled using the advection-diffusion-reaction equation:

(11) $$\vec{v}\;\cdot \;\nabla P_{{CO}_{2}}=D_{eff,{CO}_{2}}\;\nabla^{2}P_{CO_{2}}+\;S_{P_{{CO}_{2}}}$$

where $P_{{CO}_{2}}$ is the carbon dioxide partial pressure (mmHg), $D_{eff,{CO}_{2}}$ is the effective diffusivity of carbon dioxide in blood ($m^{2}/s$), and $S_{P_{{CO}_{2}}}$ is the source term for carbon dioxide partial pressure (mmHg/s). The effective diffusivity and source term are defined in Equations (12) and (13), respectively. The derivation of these expressions is provided in Appendix B.

(12) $$D_{eff,{CO}_{2}}=\frac{D_{{CO}_{2}}\;\alpha_{CO_{2}}+D_{HbCO_{2}}k_{CO_{2}}\;Hb\frac{\partial S_{{CO}_{2}}}{\partial P_{CO_{2}}}+D_{HCO_{3}^{-}}\frac{\partial C_{HCO_{3}^{-}}}{\partial P_{CO_{2}}}}{\alpha_{CO_{2}}+k_{{CO}_{2}}\;Hb\frac{\partial S_{{CO}_{2}}}{\partial P_{{CO}_{2}}}+\frac{\partial C_{HCO_{3}^{-}}}{\partial P_{CO_{2}}}}$$

(13) $$S_{P_{CO_{2}}}=\frac{D_{Hb{CO}_{2}}k_{CO_{2}}\;Hb\frac{\partial^{2}S_{CO_{2}}}{\partial P_{CO_{2}}^{2}}+D_{HCO_{3}^{-}}\;\frac{\partial^{2}C_{HCO_{3}^{-}}}{\partial P_{CO_{2}}^{2}}\;}{\alpha_{CO_{2}}+k_{{CO}_{2}}\;Hb\frac{\partial S_{{CO}_{2}}}{\partial P_{{CO}_{2}}}+\frac{\partial C_{HCO_{3}^{-}}}{\partial P_{CO_{2}}}}\;|\nabla P_{O_{2}}{|}^{2}$$

In the above equations, $D_{{CO}_{2}}$ is the diffusivity of carbon dioxide in blood ($m^{2}/s$), $\alpha_{{CO}_{2}}$ is the solubility of carbon dioxide in blood ($mL_{CO_{2}}/mL_{\mathrm{blood}}/\mathrm{mmHg}$), $D_{Hb{CO}_{2}}$ is the diffusivity of carbaminohemoglobin ($m^{2}/s$), $k_{{CO}_{2}}$ is the carbon dioxide-carrying capacity of hemoglobin ($mL_{CO_{2}}/g_{\mathrm{Hb}}$), $S_{CO_{2}}$ is the carbon dioxide saturation of hemoglobin (dimensionless), $D_{HCO_{3}^{-}}$ is the diffusivity of bicarbonate (${\mathrm{HCO}}_{3}^{-}$) in blood ($m^{2}/s$), and $C_{HCO_{3}^{-}}$ is the concentration of carbon dioxide stored in the form of bicarbonate ($mL_{CO_{2}}/mL_{\mathrm{blood}}$). Physical constants of the mentioned variables are also given in Table 1. This study calculated and implemented the effective diffusivities of oxygen and carbon dioxide in blood using user-defined functions (UDFs).

Bicarbonate acts as a physiological buffer, neutralizing the acidic effects of dissolved carbon dioxide in plasma, maintaining blood pH within a safe and stable range. The relation between carbon dioxide partial pressure ($P_{{CO}_{2}}$ in mmHg), bicarbonate concentration ($C_{HCO_{3}^{-}}$ in $\mathrm{mmol}/L_{\mathrm{blood}}$), and pH is expressed via the Henderson–Hasselbach equation [36]:

(14) $$pH=pK_{a}+\log_{10}\frac{C_{HCO_{3}^{-}}}{\alpha_{CO_{2}}\;P_{CO_{2}}}$$

In this Equation, $pK_{a}$ represents the negative logarithm of the acid dissociation constant ($K_{a}$), where a lower $pK_{a}$ indicates a stronger acid. In Equation (14), $\alpha_{CO_{2}}$, the solubility of carbon dioxide in blood is given as 0.0307 $\mathrm{mmol}/L_{\mathrm{blood}}/\mathrm{mmHg}$. An additional relationship between pH and $C_{HCO_{3}^{-}}$ is given by the buffer line equation, also known as the Van Slyke equation [37]:

(15) $$C_{HCO_{3}^{-}}=24.4-\;\beta_{NC}\times (pH-7.4)$$

(16) $$\beta_{NC}=1.43\times Hb\times 100+7.7$$

where $\beta_{NC}$ is the non-carbonic buffer power of blood, calculated using Equation (16) [37,38]. Equation (15) represents a rearranged form of the Van Slyke buffer line equation, assuming the base excess (BE) is zero at a pH of 7.4.

The pH can be calculated at a specified carbon dioxide partial pressure by solving Equations (14)–(16) using a numerical method such as the Newton–Raphson method. Consequently, the bicarbonate concentration in $\mathrm{mmol}/L_{\mathrm{blood}}$ can be determined. A factor of 0.02226 is needed to multiply by the $C_{HCO_{3}^{-}}$ to achieve it in $mL_{CO_{2}}/mL_{\mathrm{blood}}$. The commonly accepted value for $pK_{a}$ is reported to be 6.09, as reported in the literature [39]. However, this study adjusted this value to 6.082 to reproduce a pH of 7.357 at a carbon dioxide partial pressure of 44 mmHg, consistent with the experimental data reported by Focke et al. [10].

Carbon dioxide saturation ($S_{CO_{2}}$) can be modeled using the Hill equation. Based on data presented by Dash and Bassingthwaighte [40], the carbon dioxide saturation curve, at a pH of 7.4, oxygen partial pressure of 100 mmHg, and temperature of 37 °C, can be described using the following form of the Hill equation:

(17) $$S_{CO_{2}\;pH=7.4,\;T=37℃,\;P_{O_{2}}=100\;\mathrm{mmHg}}=\frac{{\left(\frac{P_{CO_{2}}}{P_{50,CO_{2}}}\right)}^{0.9942}}{1+\;{\left(\frac{P_{CO_{2}}}{P_{50,CO_{2}}}\right)}^{0.9942}}$$

where $P_{50,CO_{2}}$ is the carbon dioxide partial pressure at which hemoglobin is 50% saturated with carbon dioxide, calculated as 265 mmHg [40]. Equation (17) was modified to incorporate the effects of blood temperature, pH, and oxygen partial pressure on carbon dioxide saturation, known as the Haldane effect. The adjusted formulation is presented in Equations (18) and (19), which were fitted to the data provided by Dash and Bassingthwaighte [40].

(18) $$P_{{CO}_{2},adj}=P_{{CO}_{2}}\times 10^{0.004\;\left(37-T\right)+1.66\;\left(pH-7.4\right)+0.02\;log(100/P_{O_{2}})\;}$$

(19) $$S_{CO_{2}}=\frac{{\left(\frac{P_{CO_{2},adj}}{P_{50,CO_{2}}}\right)}^{0.9942}}{1+{\left(\frac{P_{CO_{2},adj}}{P_{50,CO_{2}}}\right)}^{0.9942}}$$

The second derivative of $C_{HCO_{3}^{-}}$ with respect to $P_{CO_{2}}$ is zero, and the second derivative of $S_{CO_{2}}$ with respect to $P_{CO_{2}}$ is considered negligible due to its low magnitude. Therefore, in this study, the source term for carbon dioxide partial pressure ($S_{P_{CO_{2}}}$) is assumed to be 0.

The carbon dioxide-carrying capacity of hemoglobin is assumed to be constant in this study. Under standard physiological conditions (pH of 7.4, temperature of 37 °C, oxygen partial pressure of 100 mmHg, carbon dioxide partial pressure of 40 mmHg, and hemoglobin concentration of 0.15 $g_{\mathrm{Hb}}/mL_{\mathrm{blood}}$), the carbon dioxide content of blood in the form of carbamino is about 0.0272 $mL_{CO_{2}}/mL_{\mathrm{blood}}$ [40]. Under these conditions, the carbon dioxide saturation is about 13.1%, from which the carbon dioxide-carrying capacity of hemoglobin is calculated to be 1.384 $mL_{CO_{2}}/g_{\mathrm{Hb}}$.

The total carbon dioxide content is calculated using the empirical mathematical Equation introduced by McHardy [28,41]. As shown in Equation (20), the carbon dioxide content ($mL_{CO_{2}}/mL_{\mathrm{blood}}$) is expressed as a function of bicarbonate concentration, hemoglobin concentration, oxygen saturation, and pH. Oxygen saturation, in turn, depends on oxygen partial pressure, blood temperature, pH, and carbon dioxide partial pressure, while bicarbonate concentration is influenced by both pH and carbon dioxide partial pressure.

(20) $$C_{CO_{2}}=C_{HCO_{3}^{-}}\;\left(1-\frac{0.2924\;Hb}{\left(2.244-0.422\;S_{O_{2}}\right)\;\left(8.74-pH\right)\;}\right)$$

Carbon dioxide transfer rate (${\dot{m}}_{{CO}_{2}}$) is calculated using Equation (21), based on the mass-weighted average of total carbon dioxide content at the inlet and outlet.

(21) $${\dot{m}}_{O_{2}}=Q_{blood}\times 10^{3}\times \left(C_{CO_{2},in}-C_{CO_{2},Out}\right)$$

For clarity, a structured summary of the model assumptions, numerical domain settings, and scope/limitations is provided in the Supplementary Materials.

### 2.5. Mesh Sensitivity Study

A mesh sensitivity study was conducted using the CFD domain with four layers of fiber mats (Figure 1c), ensuring that the simulation results were independent of mesh resolution and that the underlying physical phenomena, such as flow behavior and gas transport, were accurately resolved. Since a structured mesh generally provides higher accuracy and lower computational cost than an unstructured mesh, a structured mesh was generated in this study for a smoother gradient, better convergence, and stability. Seven structured mesh configurations were generated, ranging from approximately 250,000 to 6 million elements.

**Figure 2:**
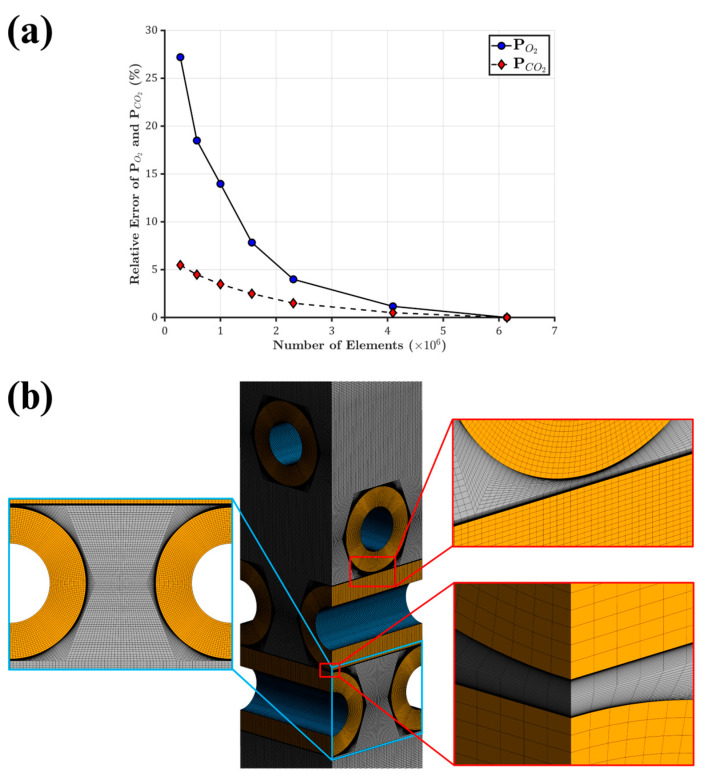
Mesh sensitivity analysis and structured mesh configuration. (**a**) Relative error of the outlet mass-weighted average oxygen and carbon dioxide partial pressures, calculated with respect to the finest mesh (6 million elements), plotted against the total number of elements. (**b**) Structured mesh used in the CFD simulations with a total of 4.1 million elements. Insets show close-up views highlighting mesh refinement around curved fiber surfaces, capturing the boundary layer, and demonstrating overall mesh quality.

Mass-weighted average of oxygen and carbon dioxide partial pressures were computed at the outlet, and the relative errors were calculated with respect to the finest mesh. As shown in Figure 2a, increasing the mesh size from 4.1 million to 6 million elements resulted in less than 2% variation in partial pressure values, indicating acceptable convergence. Therefore, the mesh with 4.1 million elements was selected for the subsequent CFD simulations, and the same settings were applied to the full oxygenator model (Figure 1d), resulting in a total mesh size of approximately 36 million. A close-up view of the 4.1 million-element mesh is shown in Figure 2b.

## 3. Results

### 3.1. Oxygen Transfer Rate

**Figure 3:**
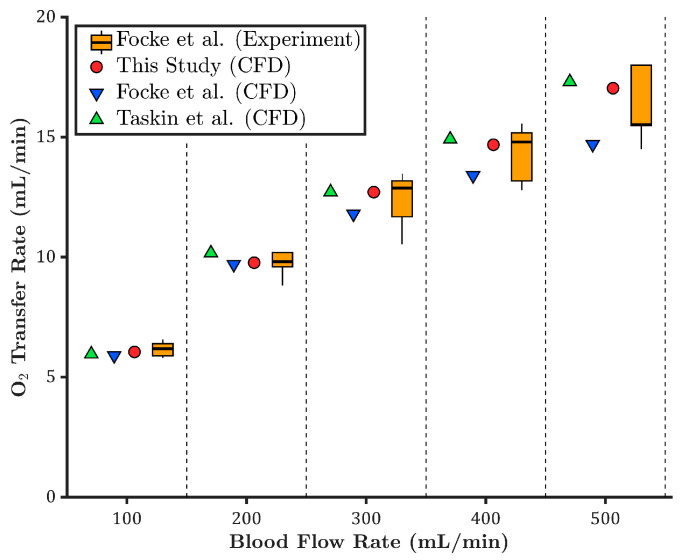
Comparison of oxygen transfer rates across different blood flow rates, based on experimental data and CFD simulations from this study, Focke et al. [10], and Taskin et al. [12].

In the study by Focke et al. [10], five independent experiments were conducted using porcine blood under venous conditions at each blood flow rate. The experimental oxygen transfer rates are presented as box plots in Figure 3. As shown, the CFD results from the present study (red circles) fall within the range of experimental values across all flow rates. Importantly, for all flow rates except 500 mL/min, the predicted oxygen transfer rates align closely with the median experimental values, indicating strong agreement and high model accuracy.

The CFD results reported by Focke et al. [10] (blue downward triangles) underpredict the oxygen transfer rate compared to the experimental data. A separate CFD analysis based on the Taskin et al. model [12] (green upward triangles) was performed, assuming a constant oxygen saturation curve. Similar to the current study, partial pressures of the inlet and inner membrane wall were adjusted to match the experimental inlet saturation and partial pressure difference.

The Taskin-based model slightly overpredicts oxygen transfer compared to the present model, likely due to excluding the Bohr effect. In contrast, the model developed in this study incorporates the Bohr effect, accounting for local variations in pH and carbon dioxide partial pressure, thereby enabling a more physiologically accurate prediction of oxygen saturation behavior and oxygen transfer rate.

**Figure 4:**
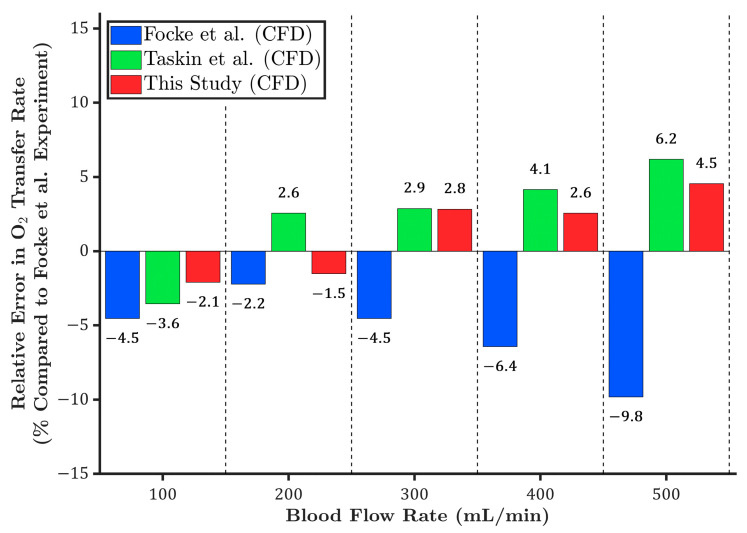
Relative error in oxygen transfer rates predicted by different CFD models compared to the mean experimental data reported by Focke et al. [10], and Taskin et al. [12] across various blood flow rates.

To better compare the CFD models, the relative error in oxygen transfer rate was calculated with respect to the mean value of the experimental data. As shown in Figure 4, the CFD results from the current study show the lowest overall relative errors, with a maximum deviation under 5% across all flow rates. The Taskin et al. [12] model demonstrates acceptable agreement, with moderate relative errors. In contrast, the CFD results by Focke et al. [10] show increasing discrepancies at higher flow rates, with the relative error reaching nearly −10% at 500 mL/min, indicating reduced accuracy in predicting oxygen transfer at high flows.

### 3.2. Carbon Dioxide Transfer Rate

**Figure 5:**
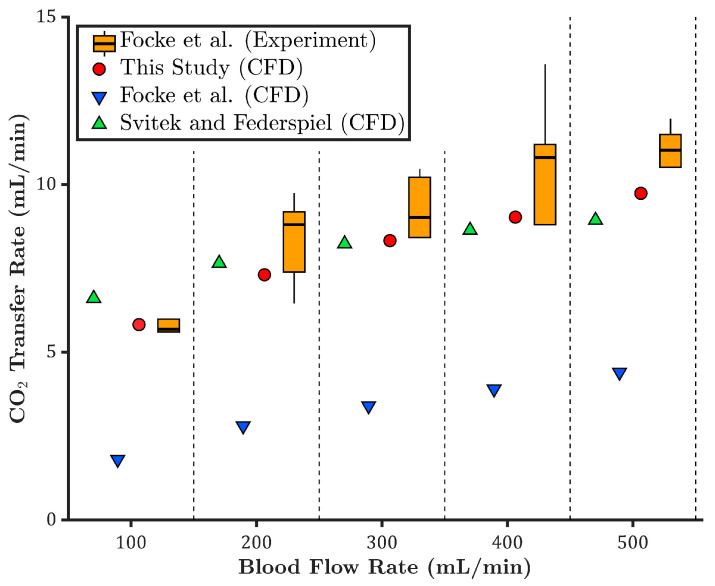
Comparison of carbon dioxide transfer rates across different blood flow rates, based on experimental data and CFD simulations from this study, Focke et al. [10], and Svitek and Federspiel [24].

Similar to the oxygen transfer rate results, the experimental carbon dioxide transfer rates are shown as box plots in Figure 5. As illustrated, the CFD results from the current study (red circles) fall within the experimental range across all flow rates except 500 mL/min, where a slight deviation from the experimental values is observed.

The CFD results reported by Focke et al. [10] (blue downward triangles) substantially underpredict the carbon dioxide transfer rate across all flow rates. This discrepancy is expected, as their model computes the carbon dioxide transfer rate based solely on the partial pressure difference of carbon dioxide. However, dissolved carbon dioxide accounts for only about 7% of the total carbon dioxide content in the blood, with the majority being transported as bicarbonate (70%) and carbaminohemoglobin (23%) [42]. Therefore, neglecting these forms of carbon dioxide transfer rate leads to a significant underestimation.

Moreover, a separate CFD analysis was conducted using the Svitek and Federspiel model [24] (green upward triangles), which assumes a constant carbon dioxide content curve with a fixed slope. As shown in Figure 5, at lower blood flow rates (100 and 200 mL/min), the Svitek and Federspiel-based model predicts higher carbon dioxide transfer rates than the present study. At 300 mL/min, the predictions from both models converge. However, as the flow rate increases to 400 and 500 mL/min, the Svitek and Federspiel model begins to underpredict the carbon dioxide transfer rate relative to the present study.

**Figure 6:**
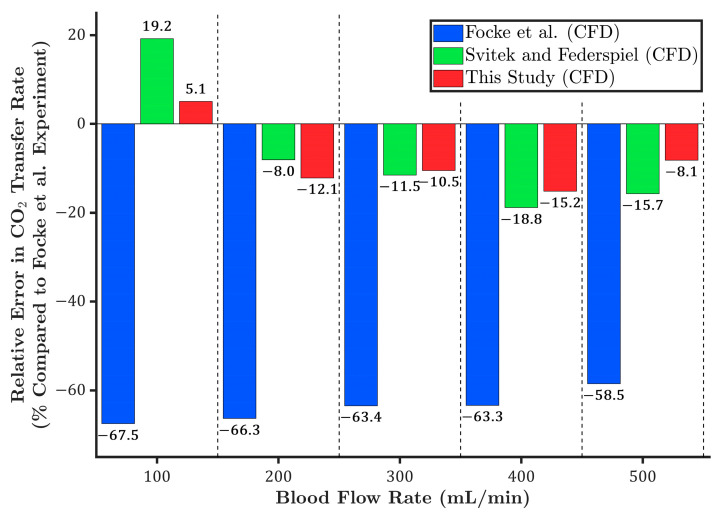
Relative error in carbon dioxide transfer rates predicted by different CFD models compared to the mean experimental data reported by Focke et al. [10], and Svitek and Federspiel [24] across various blood flow rates.

Similarly, for a deeper comparison, the relative error in carbon dioxide transfer rate was calculated with respect to the mean value of the experimental data. As shown in Figure 6, the CFD results from the present study show the lowest overall relative errors, with a maximum deviation of approximately 15%, and an average deviation of around 10%. The Svitek and Federspiel model shows better agreement at moderate flow rates but tends to overpredict carbon dioxide transfer at low flow rates and underpredict it at higher flow rates. This discrepancy might be due to the omission of physiological interactions, known as the Haldane effect, which describes the influence of pH and oxygen saturation on carbon dioxide transport and is accounted for in the present study.

As discussed earlier, the high relative error in the CFD results reported by Focke et al. [10] is attributed to their simplified model, which calculated carbon dioxide content based solely on dissolved CO_2_, neglecting the contributions of bicarbonate and hemoglobin-bound forms.

### 3.3. Comparison of Local Gas Transport Predictions with Existing Models

To further evaluate the proposed model against conventional approaches, oxygen transport was compared with the Taskin et al. [12] model, and carbon dioxide transport with the Svitek and Federspiel [24] model. Oxygen and carbon dioxide content profiles were calculated and plotted along a line defined by the intersection of the two mid-planes, for the 2nd- and 42nd-layer fibers, at blood flow rates of 100, 300, and 500 mL/min.

**Figure 7:**
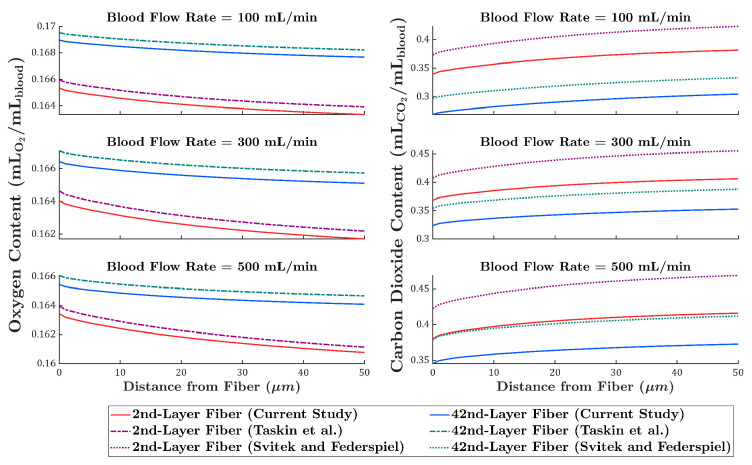
Comparison of oxygen content (**left**) and carbon dioxide content (**right**) profiles predicted by the current CFD model, the Taskin et al. [12] model (oxygen), and the Svitek and Federspiel [24] model (carbon dioxide).

As shown in Figure 7, the Taskin model consistently overpredicts oxygen content, with the difference relative to the present study remaining nearly constant across both fiber layers and all flow rates. In contrast, the Svitek and Federspiel model overpredicts carbon dioxide content, increasing the discrepancy at higher flow rates. At 500 mL/min, the predicted carbon dioxide content for the 42nd-layer fiber in their model approaches the carbon dioxide content of the 2nd-layer fiber in the present study, indicating a pronounced deviation in transport behavior.

**Figure 8:**
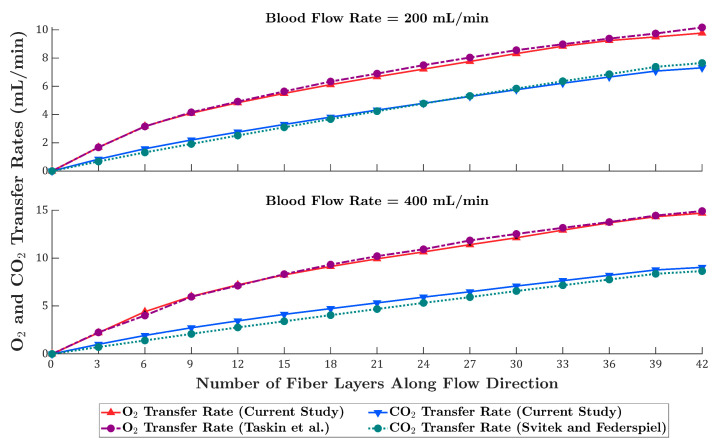
Oxygen and carbon dioxide transfer rates across fiber layers for blood flow rates of 200 mL/min (**top**) and 400 mL/min (**bottom**). Results from the current study are compared with predictions from the Taskin et al. [12] model (O_2_) and the Svitek and Federspiel [24] model (CO_2_).

Oxygen and carbon dioxide transfer rate profiles across the fiber layers for blood flow rates of 200 and 400 mL/min are presented in Figure 8. The Taskin model predicts slightly higher oxygen transfer rates at both flow rates than the current study for nearly all fiber layers. For carbon dioxide, the Svitek and Federspiel model at 200 mL/min underpredicts the transfer rate up to approximately the 27th layer, after which it overpredicts. At 400 mL/min, this model underpredicts carbon dioxide transfer across all fiber layers.

## 4. Discussion

This study introduced and validated a novel CFD model incorporating the Bohr and Haldane effects. The proposed model demonstrated strong agreement with the experimental data from Focke et al. [10] for both oxygen and carbon dioxide transfer rates across all tested flow rates ranging from 100 to 500 mL/min. The relative error of the current CFD model with the experimental mean values remained below 5% for oxygen transfer rate and approximately 10–15% for carbon dioxide transfer rate. Compared to existing models, Focke et al. [10], Taskin et al. [12], and Svitek and Federspiel [24], the proposed model consistently showed improved predictive accuracy. A slight reduction in the model accuracy was observed at the highest flow rate, which can be attributed to steeper partial pressure gradients. Nevertheless, the overall level of agreement indicates that the model captures the underlying coupled gas exchange mechanisms robustly.

The selected blood flow range of 100–500 mL/min was chosen to match the experimental data of Focke et al. [10], corresponding to Reynolds numbers between 0.7 and 3.5. Although these values are lower than typical clinical flow rates (1–5 mL/min), the associated superficial velocities in our study (3.4–17 mm/s) closely overlap with those in commercial oxygenators. For example, according to the Adult OXY1 oxygenator specifications reported by Zhang et al. [19], blood flow rates of 1–5 mL/min translate to the superficial velocity of 2.35–11.75 mm/s. Thus, the simulated flow regime is representative of clinically relevant operating conditions, ensuring the physiological applicability of the results.

The results show that the oxygen transfer rate predicted by the Taskin model closely matches that of the current model, which incorporates the Bohr effect. This similarity can be attributed to two main factors. First, the oxygen diffusivity used in this study is very similar to that in the Taskin model, as the diffusion coefficient of oxyhemoglobin is negligible and can be disregarded. Second, while the Bohr effect does shift the oxygen saturation curve, the magnitude of this shift is relatively small, resulting in only minor differences in the predicted oxygen transfer rates between the two models.

Unlike previous CFD models that neglected the coupled interaction between oxygen and carbon dioxide transport, this study presents a physiologically informed approach that captures these interdependencies. In the proposed CFD model, oxygen saturation, carbon dioxide saturation, and total carbon dioxide content are expressed as functions of oxygen and carbon dioxide partial pressures, blood temperature, and pH. The aforementioned coupled interaction, representing the Bohr and Haldane effects, enables a more accurate depiction of local gas exchange dynamics, especially near the fiber walls with steep gradients.

The findings of this study offer valuable insights into the design and simulation of hollow fiber membrane oxygenators. Physiological interactions between oxygen and carbon dioxide transport, which are overlooked in CFD simulations and critically affect gas exchange, can be captured by incorporating the Bohr and Haldane effects. Such detailed modeling provides a more accurate prediction of oxygen and carbon dioxide transfer, which can significantly reduce reliance on labor-intensive, time-consuming, and costly in vitro testing during the device development phase. Beyond improving predictive accuracy, this framework offers practical implications for oxygenator design and performance optimization. By resolving local variations in gas transfer, the model can help identify fiber arrangements (similar to our previous study [9]), bundle porosities, and operating conditions that maximize oxygen uptake while ensuring efficient carbon dioxide removal. Furthermore, as blood flow rates increase, steep gradients in partial pressures and pH become more noticeable, making it essential to include these physiological effects for high-fidelity modeling. The proposed CFD model can be a foundation for optimizing next-generation oxygenators and enabling patient-specific simulations or personalized oxygenator design.

Various two-phase modeling approaches for the hollow fiber membranes have been reported in previous studies [10,11,13,18,22]. Although Poletti et al. [13] validated their multiphase CFD model with experimental data, selecting an appropriate multiphase framework and interphase momentum exchange model (such as drag models) remains a significant challenge. Moreover, two-phase modeling substantially increases computational complexity, often leading to convergence difficulties. In contrast, when combined with properly defined effective diffusivities, the single-phase approach used in the present study demonstrated comparable and superior agreement with experimental results.

The findings also suggest that incorporating a small gap between fibers may be a more practical and effective approach than modeling direct fiber contacts or rounding sharp edges, as done in some previous studies [10,13]. Modeling fiber contacts increases meshing complexity and typically requires unstructured meshes with a significantly larger number of elements. In contrast, introducing a small gap allows for structured meshing, reduces the element count, improves mesh quality, and computational efficiency.

In the Svitek and Federspiel study [24], the total carbon dioxide content curve was assumed to be constant with a fixed slope over a partial pressure range of 25 to 50 mmHg. However, multiple CFD simulations have demonstrated that the partial pressure of carbon dioxide near the fiber walls can fall below this range, rendering the constant slope assumption invalid. Omecinski and Federspiel [28] extended this model by incorporating the Haldane effect into the mathematical formulation. However, their model assumed a constant blood pH, oversimplifying physiological conditions, while Khadka et al. [29] showed that carbon dioxide removal leads to a monotonic increase in pH during ECMO.

Furthermore, in the study by Svitek and Federspiel [24], the derivation of the effective carbon dioxide diffusivity involved dividing the diffusive transport terms by the convective transport terms, which included the derivatives of dissolved CO_2_ and bound CO_2_ (bicarbonate and hemoglobin-bound) with respect to CO_2_ partial pressure. While the convective terms should represent the derivative of the total CO_2_ content, they assumed that the derivative of dissolved CO_2_ is equal to the CO_2_ solubility, and that the derivative of bound CO_2_ is approximately equal to the derivative of the total CO_2_ content. As a result, in their formulation of the derivative of the convective term with respect to CO_2_ partial pressure, the CO_2_ solubility is effectively added, leading to an underestimation of the effective CO_2_ diffusivity.

The present model was developed under the steady-state flow conditions. However, this assumption is consistent with most CFD studies on membrane oxygenators, where steady-state simulations have served as a practical and reliable foundation for model development and validation. Nonetheless, clinical ECMO circuits frequently operate under pulsatile or time-varying flow, and extending the model to account for such dynamics remains an important direction for future research. In principle, the current framework can be adapted to transient CFD simulations, allowing investigation of pulsatility effects on local gas transfer and flow patterns, as demonstrated in preliminary work by Tang et al. [43].

While the model demonstrates improved accuracy in predicting oxygen and carbon dioxide transfer, it employs a single-phase blood model. Therefore, microscale phenomena such as non-uniform hematocrit distribution, cell-free layer formation near the walls [23], or shear-induced migration of red blood cells cannot be captured. Additionally, the current study was limited to one geometry; therefore, broader validation with different oxygenator types is necessary to confirm the model’s generalizability. However, the framework relies on fundamental transport equations coupled with physiologically informed diffusivities, and can be readily applied to alternative geometries, fiber arrangements, or commercial oxygenators. Extending the proposed model to include clot formation [16,17] and hemolysis [9,44] could also enhance its utility for evaluating device safety.

## 5. Conclusions

For the first time, a CFD gas transfer model incorporating physiologically relevant gas exchange interactions, specifically the Bohr and Haldane effects, was developed and validated with experimental data from Focke et al. [10]. The model demonstrated improved predictive accuracy for oxygen and carbon dioxide transfer rates across a range of flow conditions. Compared to previous models, the proposed approach more effectively captures the coupled dynamics of gas transport, particularly near the fiber walls, where steep gradients dominate mass transfer.

The results indicated that the CFD study by Focke et al. [10] significantly underestimated the carbon dioxide transfer rate due to oversimplified assumptions in its transport model. In contrast, the CFD simulations based on the Svitek and Federspiel [24] model showed reasonable agreement at moderate blood flow rates. Still, they failed to accurately predict the carbon dioxide transfer rate at low and high extremes. The CFD results derived from Taskin et al.’s [12] model closely matched those of the current study, suggesting that the Haldane effect has a more substantial quantitative influence on carbon dioxide transport than the Bohr effect has on oxygen transport.

The results also show that the oxygen content in the Taskin model is slightly overpredicted, whereas the carbon dioxide content in the Svitek and Federspiel model is substantially overpredicted, particularly at higher blood flow rates.

The proposed model offers a computationally efficient and physiologically accurate tool for oxygenator design and optimization, enhancing realism without the need for complex multiphase modeling. Although this study focused on a single geometry under steady-state conditions, the model provides a strong foundation for future extensions. It can be adapted to incorporate pulsatile flow, diverse oxygenator configurations, and additional clinical parameters to further support device development and patient-specific simulations.

## Figures and Tables

**Table 1:** Physical constants used in the oxygen and carbon dioxide transport models.

Property	Symbol	Value	Unit
Oxygen diffusion coefficient in blood	$D_{O_{2}}$	$1.9\times 10^{-9}$ [12]	$m^{2}/s$
Oxygen solubility in blood	$\alpha_{O_{2}}$	$3\times 10^{-5}$ [12]	$mL_{O_{2}}/mL_{blood}/mmHg$
Oxyhemoglobin diffusion coefficient in blood	$D_{HbO_{2}}$	$1.44\times 10^{-11}$ [18]	$m^{2}/s$
Oxygen-carrying capacity of hemoglobin	$k_{O_{2}}$	1.34 [12]	$mL_{O_{2}}/g_{Hb}$
Total hemoglobin concentration	Hb	0.118 [10]	$g_{Hb}/{mL}_{blood}$
Carbon dioxide diffusion coefficient in blood	$D_{{CO}_{2}}$	$7.39\times 10^{-10}$ [24]	$m^{2}/s$
Carbon dioxide solubility in blood	$\alpha_{{CO}_{2}}$	0.0307 [29]	$mmol/L_{blood}/mmHg$
Carbaminohemoglobin diffusion coefficient in blood	$D_{{HbCO}_{2}}$	$1.44\times 10^{-11}$	$m^{2}/s$
Carbon dioxide-carrying capacity of hemoglobin	$k_{CO_{2}}$	1.384	$mL_{{CO}_{2}}/g_{Hb}$
Bicarbonate diffusion coefficient in blood	$D_{HCO_{3}^{-}}$	$4.62\times 10^{-10}$ [24]	$m^{2}/s$

## Data Availability

The original contributions presented in this study are included in the article and Supplementary Materials. Further inquiries can be directed to the corresponding author.

## Supplementary Materials

The following supporting information can be downloaded at: https://www.mdpi.com/article/10.3390/membranes15090268/s1, including details on model assumptions, numerical domain and simulation settings, scope and limitations. References [10,12,24] are cited in the supplementary materials.

## Appendix A

Oxygen Transport Equations

Oxygen in blood exists in 2 forms:

1. Dissolved oxygen in plasma

2. Oxygen bound to hemoglobin

Accordingly, the convection–diffusion–reaction equations governing these two forms of oxygen transport are as follows:

(A1) $$\vec{v}\;\cdot \;\nabla \left(C_{O_{2}}^{d}\right)=D_{O_{2}}\;\nabla^{2}\left(C_{O_{2}}^{d}\right)-R$$

(A2) $$\vec{v}\;\cdot \;\nabla \left(C_{O_{2}}^{b}\right)=D_{{HbO}_{2}}\;\nabla^{2}\left(C_{O_{2}}^{b}\right)+R$$

The concentrations of dissolved oxygen in plasma and oxygen bound to hemoglobin can be expressed as:

(A3) $$C_{O_{2}}^{d}=\alpha_{O_{2}}P_{O_{2}}$$

(A4) $$C_{O_{2}}^{b}=k_{O_{2}}Hb{\;S}_{O_{2}}$$

where $\alpha_{O_{2}}$ is the solubility of oxygen in blood in $mL_{O_{2}}/mL_{\mathrm{blood}}/\mathrm{mmHg}$, $P_{O_{2}}$ is the oxygen partial pressure in mmHg, $k_{O_{2}}$ is the binding capacity of oxygen to hemoglobin (Hüfner coefficient) in $mL_{O_{2}}{/g}_{\mathrm{Hb}}$, Hb is the total hemoglobin concentration in $g_{\mathrm{Hb}}/mL_{\mathrm{blood}}$, and ${\;S}_{O_{2}}$ is the oxygen saturation. First, R is calculated from Equation (A2):

(A5) $$R=\;\vec{v}\;\cdot \;\nabla \left(k_{O_{2}}\;Hb{\;S}_{O_{2}}\right)-\;D_{{HbO}_{2}}\;\nabla^{2}\left(k_{O_{2}}\;Hb{\;S}_{O_{2}}\right)$$

The Hüfner coefficient and hemoglobin concentration are assumed to be constant, while oxygen saturation is expressed as a function of the oxygen partial pressure, blood temperature, pH, and carbon dioxide partial pressure. Accordingly, the gradients in Equation (A5) can be expressed as:

(A6) $$\vec{v}\;\cdot \;\nabla \left(k_{O_{2}}\;Hb{\;S}_{O_{2}}\right)=\;k_{O_{2}}\;Hb\frac{\partial S_{O_{2}}}{\partial P_{O_{2}}}\;\vec{v}\cdot \nabla P_{O_{2}}$$

(A7) $$\nabla^{2}\left(k_{O_{2}}\;Hb{\;S}_{O_{2}}\right)=k_{O_{2}}\;Hb\;\left(\frac{\partial S_{O_{2}}}{\partial P_{O_{2}}}\;\nabla^{2}P_{O_{2}}+\frac{\partial^{2}S_{O_{2}}}{\partial P_{O_{2}}^{2}}|\nabla P_{O_{2}}{|}^{2}\right)$$

Substitute Equations (A5)–(A7) to A1, and rearrange terms:

(A8) $$\left[\alpha_{O_{2}}+k_{O_{2}}Hb\frac{\partial S_{O_{2}}}{\partial P_{O_{2}}}\right]\vec{v}.\nabla P_{O_{2}}\;\;\;\;\;\;\;\;\;\;\;\;\;\;\;\;\;\;=\left[D_{O_{2}}\alpha_{O_{2}}+D_{HbO_{2}}k_{O_{2}}Hb\frac{\partial S_{O_{2}}}{\partial P_{O_{2}}}\right]\nabla^{2}P_{O_{2}}\;\;\;\;\;\;\;\;\;\;\;\;\;\;\;+\left[D_{HbO_{2}}k_{O_{2}}Hb\frac{\partial^{2}S_{O_{2}}}{\partial P_{O_{2}}^{2}}\right]|\nabla P_{O_{2}}{|}^{2}$$

By dividing the right-hand side of Equation (A8) by $\alpha_{O_{2}}+k_{O_{2}}\;Hb\frac{\partial S_{O_{2}}}{\partial P_{O_{2}}}$, the following convection–diffusion–reaction equation is obtained:

(A9) $$\vec{v}\;\cdot \;\nabla P_{O_{2}}=D_{eff,O_{2}}\;\nabla^{2}P_{O_{2}}+\;S_{P_{O_{2}}}$$

where $D_{eff,O_{2}}$ and $S_{P_{O_{2}}}$ are defined in Equations (A10) and (A11), respectively.

(A10) $$D_{eff,O_{2}}=\frac{D_{O_{2}}\;\alpha_{O_{2}}+D_{HbO_{2}}k_{O_{2}}\;Hb\frac{\partial S_{O_{2}}}{\partial P_{O_{2}}}}{\alpha_{O_{2}}+k_{O_{2}}\;Hb\frac{\partial S_{O_{2}}}{\partial P_{O_{2}}}}$$

(A11) $$S_{P_{O_{2}}}=\frac{D_{HbO_{2}}k_{O_{2}}\;Hb\frac{\partial^{2}S_{O_{2}}}{\partial P_{O_{2}}^{2}}\;}{\alpha_{O_{2}}+k_{O_{2}}\;Hb\frac{\partial S_{O_{2}}}{\partial P_{O_{2}}}}\;|\nabla P_{O_{2}}{|}^{2}$$

Assuming $D_{HbO_{2}}=0$ reduces the model to that proposed by Taskin et al. [12].

## Appendix B

Carbon Dioxide Transport Equations

Carbon dioxide in blood exists in 3 forms:

1. Dissolved carbon dioxide in plasma

2. Bicarbonate (HCO_3_^−^)

3. Carbaminohemoglobin

Accordingly, the convection–diffusion–reaction equations governing these two forms of oxygen transport are as follows:

(A12) $$\vec{v}\;\cdot \;\nabla \left(C_{{CO}_{2}}^{d}\right)=D_{{CO}_{2}}\;\nabla^{2}\left(C_{{CO}_{2}}^{d}\right)+R_{1}+R_{2}$$

(A13) $$\vec{v}\;\cdot \;\nabla \left(C_{{CO}_{2}}^{b}\right)=D_{{HbCO}_{2}}\;\nabla^{2}\left(C_{{CO}_{2}}^{b}\right)-R_{1}$$

(A14) $$\vec{v}\;\cdot \;\nabla \left(C_{HCO_{3}^{-}}\right)=D_{HCO_{3}^{-}}\;\nabla^{2}\left(C_{HCO_{3}^{-}}\right)-R_{2}$$

The concentrations of dissolved carbon dioxide in plasma and carbon dioxide bound to hemoglobin can be expressed as:

(A15) $$C_{{CO}_{2}}^{d}=\;\alpha_{{CO}_{2}}\;P_{{CO}_{2}}$$

(A16) $$C_{{CO}_{2}}^{b}=k_{{CO}_{2}}\;Hb{\;S}_{{CO}_{2}}$$

Similarly, $\alpha_{CO_{2}}$ is the solubility of carbon dioxide in blood in $mL_{{\mathrm{CO}}_{2}}/mL_{\mathrm{blood}}/\mathrm{mmHg}$, $P_{{CO}_{2}}$ is the carbon dioxide partial pressure in mmHg, $k_{CO_{2}}$ is the binding capacity of carbon dioxide to hemoglobin in $mL_{{\mathrm{CO}}_{2}}{/g}_{\mathrm{Hb}}$, Hb is the total hemoglobin concentration in $g_{\mathrm{Hb}}/mL_{\mathrm{blood}}$, and ${\;S}_{CO_{2}}$ is the carbon dioxide saturation.

$R_{1}$ is calculated from Equation (A13):

(A17) $$R_{1}=\;\vec{v}\;\cdot \;\nabla \left(k_{{CO}_{2}}\;Hb{\;S}_{{CO}_{2}}\right)-\;D_{{HbCO}_{2}}\;\nabla^{2}\left(k_{CO_{2}}\;Hb{\;S}_{{CO}_{2}}\right)$$

Similarly, $R_{2}$ is calculated from Equation (A14):

(A18) $$R_{2}=\;\vec{v}\;\cdot \;\nabla \left(C_{HCO_{3}^{-}}\right)-\;D_{HCO_{3}^{-}}\;\nabla^{2}\left(C_{{\mathrm{HCO}}_{3}^{-}}\right)$$

Carbon dioxide saturation is modeled as a function of carbon dioxide partial pressure, pH, and oxygen partial pressure, while bicarbonate concentration is expressed as a function of carbon dioxide partial pressure and pH. Therefore, the gradients in Equations (A17) and (A18) can be written as:

(A19) $$\vec{v}\;\cdot \;\nabla \left(k_{{CO}_{2}}\;Hb{\;S}_{{CO}_{2}}\right)=\;k_{{CO}_{2}}\;Hb\frac{\partial S_{CO_{2}}}{\partial P_{CO_{2}}}\;\vec{v}.\nabla P_{CO_{2}}$$

(A20) $$\nabla^{2}\left(k_{{CO}_{2}}\;Hb{\;S}_{{CO}_{2}}\right)=k_{{CO}_{2}}\;Hb\;\left(\frac{\partial S_{CO_{2}}}{\partial P_{CO_{2}}}\;\nabla^{2}P_{CO_{2}}+\frac{\partial^{2}S_{CO_{2}}}{\partial P_{CO_{2}}^{2}}|\nabla P_{CO_{2}}{|}^{2}\right)$$

(A21) $$\vec{v}\;\cdot \;\nabla \left(C_{HCO_{3}^{-}}\right)=\frac{\partial C_{HCO_{3}^{-}}}{\partial P_{CO_{2}}}\;\vec{v}\cdot \nabla P_{CO_{2}}$$

(A22) $$\nabla^{2}\left(C_{HCO_{3}^{-}}\right)=\frac{\partial C_{HCO_{3}^{-}}}{\partial P_{CO_{2}}}\;\nabla^{2}P_{CO_{2}}+\frac{\partial^{2}C_{HCO_{3}^{-}}}{\partial P_{CO_{2}}^{2}}|\nabla P_{CO_{2}}{|}^{2}$$

Substitute Equations (A19)–(A22) to (A12), and rearrange terms:

(A23) $$\left[\alpha_{CO_{2}}+k_{{CO}_{2}}\;Hb\frac{\partial S_{{CO}_{2}}}{\partial P_{{CO}_{2}}}+\frac{\partial C_{HCO_{3}^{-}}}{\partial P_{CO_{2}}}\right]\;\vec{v}\;.\;\nabla P_{CO_{2}}\;\;\;\;\;\;\;\;\;\;\;\;\;\;\;\;\;\;\;\;\;=\left[D_{{CO}_{2}}\;\alpha_{CO_{2}}+D_{HbCO_{2}}k_{CO_{2}}\;Hb\frac{\partial S_{{CO}_{2}}}{\partial P_{CO_{2}}}+D_{HCO_{3}^{-}}\frac{\partial C_{HCO_{3}^{-}}}{\partial P_{CO_{2}}}\right]\;\nabla^{2}P_{CO_{2}}\;\;\;\;\;\;\;\;\;\;\;\;\;\;\;\;\;\;+\left[D_{Hb{CO}_{2}}k_{CO_{2}}\;Hb\frac{\partial^{2}S_{CO_{2}}}{\partial P_{CO_{2}}^{2}}+D_{HCO_{3}^{-}}\;\frac{\partial^{2}C_{HCO_{3}^{-}}}{\partial P_{CO_{2}}^{2}}\;\right]\;|\nabla P_{{CO}_{2}}{|}^{2}$$

By dividing the right-hand side of Equation (A23) by $\alpha_{CO_{2}}+k_{{CO}_{2}}\;Hb\frac{\partial S_{{CO}_{2}}}{\partial P_{{CO}_{2}}}+\frac{\partial C_{HCO_{3}^{-}}}{\partial P_{CO_{2}}}$, the following convection–diffusion–reaction equation is obtained:

(A24) $$\vec{v}\;\cdot \;\nabla P_{CO_{2}}=D_{eff,{CO}_{2}}\;\nabla^{2}P_{CO_{2}}+\;S_{P_{{CO}_{2}}}$$

where $D_{eff,{CO}_{2}}$ and $S_{P_{CO_{2}}}$ are defined in Equations (A25) and (A26), respectively.

(A25) $$D_{eff,{CO}_{2}}=\frac{D_{{CO}_{2}}\;\alpha_{CO_{2}}+D_{HbCO_{2}}k_{CO_{2}}\;Hb\frac{\partial S_{{CO}_{2}}}{\partial P_{CO_{2}}}+D_{HCO_{3}^{-}}\frac{\partial C_{HCO_{3}^{-}}}{\partial P_{CO_{2}}}}{\alpha_{CO_{2}}+k_{{CO}_{2}}\;Hb\frac{\partial S_{{CO}_{2}}}{\partial P_{{CO}_{2}}}+\frac{\partial C_{HCO_{3}^{-}}}{\partial P_{CO_{2}}}}$$

(A26) $$S_{P_{CO_{2}}}=\frac{D_{Hb{CO}_{2}}k_{CO_{2}}\;Hb\frac{\partial^{2}S_{CO_{2}}}{\partial P_{CO_{2}}^{2}}+D_{HCO_{3}^{-}}\;\frac{\partial^{2}C_{HCO_{3}^{-}}}{\partial P_{CO_{2}}^{2}}\;}{\alpha_{CO_{2}}+k_{{CO}_{2}}\;Hb\frac{\partial S_{{CO}_{2}}}{\partial P_{{CO}_{2}}}+\frac{\partial C_{HCO_{3}^{-}}}{\partial P_{CO_{2}}}}\;|\nabla P_{CO_{2}}{|}^{2}$$
