# Supplementary material for: A Validated CFD Model for Gas Exchange in Hollow Fiber Membrane Oxygenators: Incorporating the Bohr and Haldane Effects
Source: Membranes (Basel). 2025 Sep 4;15(9):268. doi: 10.3390/membranes15090268 (PMC12471639; doi:10.3390/membranes15090268)
Supplement: Supplementary file 1 [file membranes-15-00268-s001.zip › membranes-3837827-supplementary.pdf]

Assumptions, Domain Settings, and Scope/Limitations

Model Assumptions

- Blood modeled as a **single-phase non-Newtonian fluid** using the Carreau–Yasuda viscosity model.
- **Steady-state flow** conditions assumed (no pulsatile or transient effects).
- **Constant hemoglobin concentration** and Hüfner coefficient applied.
- Neglected **fiber-to-fiber contacts** by introducing a small uniform gap of 10 μm for structured meshing.
- CO_2 carrying capacity of hemoglobin assumed constant under standard physiological conditions.
- Thermal effects neglected; temperature fixed at **37 °C (310 K)**.
- Periodic boundary condition on the side walls assumed.

Numerical Domain and Simulation Settings

- Oxygenator geometry based on **Focke et al.**, with 42 layers of stacked fiber mats in staggered configuration, representing the full length of 16 mm.
- Fiber dimensions: **380 μm outer diameter, 200 μm inner diameter**.
- Velocity inlet boundary condition with superficial blood velocities corresponding to flow rates of **100–500 mL/min** (Re = 0.7–3.5).
- Zero pressure outlet
- Gas diffusion in the membrane modeled explicitly using **Knudsen diffusion** with permeation values from literature.
- Inlet partial pressure of 35.9 mmHg for oxygen to satisfy the 65% oxygen saturation using Kelman subroutine
- Inner fiber wall pressure of 707.7 mmHg for oxygen to match the partial pressure difference with literature
- Inlet partial pressure of 44 mmHg for CO_2 and inner fiber wall pressure of 0
- UDFs used for diffusivity of O_2 and CO_2 in blood
- The SIMPLE algorithm used for pressure–velocity coupling

- Discretizing pressure using the PRESTO scheme and momentum and UDS equations using the QUICK scheme.

- A convergence criterion of 10^{-5} was set for all variables.

- Kelman subroutine formula for oxygen saturation and Hill equation for carbon dioxide saturation curves

- Mesh sensitivity analysis performed; final domain discretized with **36 million elements** using structured meshing.

Scope and Limitations

- Model validated against **in vitro data from Focke et al.** and compared with existing CFD approaches (Taskin et al., Svitek and Federspiel).
- Applicable to **laminar flow regimes** with superficial velocities similar to those of commercial oxygenators.
- Accuracy shown to be <5% error for O_2 and ~10–15% error for CO_2 within the tested flow range.
- Validation limited to **one oxygenator geometry**; broader generalization requires future experimental datasets.
- Does not capture microscale phenomena such as **RBC migration, cell-free layer formation, hemolysis, or clotting**.
- Transient (pulsatile) flow conditions not included; potential future extension.

References

1. Focke, J.M.; Bonke, P.-L.; Gendron, N.; Call, T.; Steinseifer, U.; Arens, J.; Neidlin, M. The influence of membrane fiber arrangement on gas exchange in blood oxygenators: A combined numerical and experimental analysis. *J. Membr. Sci.* **2024**, *710*, 123147.
2. Taskin, M.E.; Fraser, K.H.; Zhang, T.; Griffith, B.P.; Wu, Z.J. Micro-scale modeling of flow and oxygen transfer in hollow-fiber membrane bundle. *J. Membr. Sci.* **2010**, *362*, 172–183.
3. Svitek, R.G.; Federspiel, W.J. A mathematical model to predict CO2 removal in hollow fiber membrane oxygenators. *Ann. Biomed. Eng.* **2008**, *36*, 992–1003
